# The Limits of Earthquake Early Warning Accuracy and Best Alerting Strategy

**DOI:** 10.1038/s41598-019-39384-y

**Published:** 2019-02-21

**Authors:** Sarah E. Minson, Annemarie S. Baltay, Elizabeth S. Cochran, Thomas C. Hanks, Morgan T. Page, Sara K. McBride, Kevin R. Milner, Men-Andrin Meier

**Affiliations:** 1 U.S. Geological Survey, Menlo Park, California, 94025 USA; 2 U.S. Geological Survey, Pasadena, California, 91106 USA; 3 University of Southern California, Los Angeles, California, 90089 USA; 4 California Institute of Technology, Pasadena, California, 91125 USA

## Abstract

We explore how accurate earthquake early warning (EEW) can be, given our limited ability to forecast expected shaking even if the earthquake source is known. Because of the strong variability of ground motion metrics, such as peak ground acceleration (PGA) and peak ground velocity (PGV), we find that correct alerts (i.e., alerts that accurately estimate the ground motion will be above a predetermined damage threshold) are not expected to be the most common EEW outcome even when the earthquake magnitude and location are accurately determined. Infrequently, ground motion variability results in a user receiving a false alert because the ground motion turned out to be significantly smaller than the system expected. More commonly, users will experience missed alerts when the system does not issue an alert but the user experiences potentially damaging shaking. Despite these inherit limitations, EEW can significantly mitigate earthquake losses for false-alert-tolerant users who choose to receive alerts for expected ground motions much smaller than the level that could cause damage. Although this results in many false alerts (unnecessary alerts for earthquakes that do not produce damaging ground shaking), it minimizes the number of missed alerts and produces overall optimal performance.

## Introduction

The goal of earthquake early warning (EEW) is to provide advance warning that expected ground motion at a user’s location will exceed the level that may result in damage, so that people and automated systems can take action to prevent that potential damage. “Damage” can be anything the user perceives it to be; it could be structural, non-structural, or even emotional. Whether the goal is to prevent a valuable piece of manufacturing equipment from being broken or to alleviate the fear that would otherwise result when someone feels shaking without advance notice, the EEW system is performing the same action: identifying that the user will experience ground motion exceeding some critical threshold (perhaps 10%*g* acceleration for the equipment and a “felt” level of shaking for the person) and alerting that user before the ground motion actually exceeds that threshold. In this paper we ask a simple question: how accurately can an ideal EEW system predict that ground shaking will exceed a particular threshold?

Most earthquake early warning systems, such as the Japan Meteorological Agency (JMA) and the United States’ ShakeAlert systems, use real-time seismic data to determine the location and magnitude of an earthquake^1,2^. This information is then input for a ground motion prediction equation^3^ (GMPE) to calculate the expected ground motion as a function of distance from the rupture. Regions for which the expected ground motion is greater than some critical threshold are then alerted. In Japan, for example, if the expected ground motion within any subprefecture is greater than JMA Intensity 4 (approximately equivalent to Modified Mercalli Intensity VI-VII), the entire subprefecture is alerted^4^.

To explore the upper limit on the accuracy of EEW alerts, we imagine that impossibly omniscient algorithms provide us instantly and accurately an earthquake’s final source properties the moment the rupture begins. (This is beyond a best-case scenario since it is not realistic to assume that an EEW system can instantaneously predict the final earthquake rupture^5^). “Perfect” knowledge of final source properties will nevertheless not prevent false and missed alerts that arise from our imperfect ability to estimate the imminently arriving ground motion for a given earthquake.

We begin by assuming the EEW system will alert the user to take action if the expected ground motion is at least as large as the level that will cause damage; we then explore what happens if the ground motion threshold that triggers an alert is set higher or lower than that level. Different users will define damage at different ground motion thresholds, depending on their specific application. And again, “damage” can be any physical or psychological outcome one would like to avoid.

**Figure 1:**
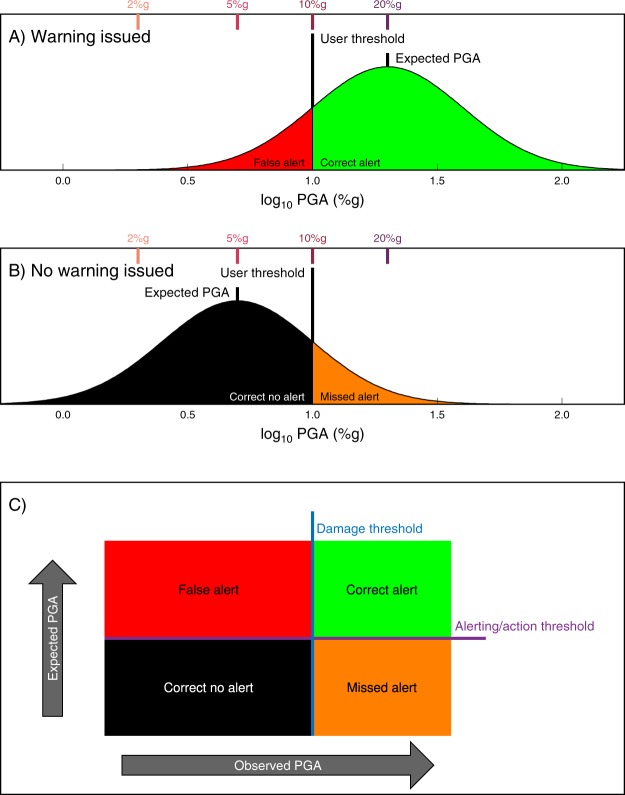
Ground motion variability and alerting accuracy. Consider a user who wishes to receive alerts for ground motion exceeding 10%*g*. In the first example, (**A**) the EEW system expects the ground motion at the user’s location to be 20%*g* and issues that user an alert. In the second example, (**B**) the expected ground motion is only 5%*g*, and thus no alert is issued. However, the ground motion calculated from a GMPE represents the median expected ground motion. Observed ground motions are typically distributed about that median with factor of two uncertainty^10^, shown by bell curve probability density functions (PDFs). By construction, in both of these examples, the EEW system will be correct for most earthquakes: (**A**) mostly results in correct alerts (green region of PDF) and (**B**) mostly results in correct no alerts (black region). However, (**A**) will result in a false alert in the unlikely case that this earthquake produces much less ground motion than expected (red region of PDF), and (**B**) will result in a missed alert for damaging ground motion in the unlikely case that this earthquake produces much stronger shaking than expected (orange region of PDF). In (**A**) and (**B**) probability is plotted as a function of log_10_ PGA, shown on the *x*-axis. The top *x*-axis calls out the four PGA thresholds we will explore in detail: 2, 5, 10, and 20%*g*. (**C**) We can alternately visualize this as a binary classification. There is some ground motion threshold above which damage is expected to occur (blue line). (This threshold was 10%*g* in (**A**) and (**B**)). The user is alerted to take action if the expected PGA is greater than some triggering value (purple line). If the expected PGA is greater than the alerting threshold, an alert will be issued (as in (**A**)) and this alert will be either correct or a false alert depending on what the observed PGA turns out to be. Likewise, if the expected PGA is less than the alerting threshold, no alert is issued (as in (**B**)) and this results in either a missed alert or the “correct no alert” case depending on the observed PGA. Note that the alerting threshold can be lower or higher than the damage threshold depending on whether the user is tolerant or intolerant of false alerts, respectively.

We adopt the following definitions^6^: a *correct alert* is when the expected ground motion exceeds a user’s threshold and the actual ground motion does as well. A *false alert* occurs when the actual ground motion falls short of that threshold. Similarly, a *correct no alert* occurs when no alert is issued because the EEW system correctly expects the ground motion will be less than the threshold. Its opposite case, a *missed alert*, occurs when the actual ground motion exceeds the user’s threshold (Fig. 1).

EEW is unlike other natural hazard warning systems in that real-time human oversight of alert issuance is impossible due to the short time period over which the earthquake rupture evolves and ground motion propagates. For most public warnings, scientists and emergency managers work together to determine what sort of alerts are necessary to protect the public from risks. However, neither scientists nor emergency managers have time to approve of a given EEW alert before it is disseminated. Thus, in addition to errors in the degree of impact (e.g., over- or under-estimating ground shaking), a real EEW system is susceptible to technical glitches, common to any automated system, such as issuing an alert when no earthquake is happening or missing an earthquake altogether. The perfect EEW system we imagine here never makes such mistakes.

Inputting the earthquake magnitude and user’s distance from the rupture into a GMPE yields the median expected peak ground acceleration (PGA), peak ground velocity (PGV), modified Mercalli intensity (MMI), spectral acceleration or other ground motion metrics. The analysis we present here is applicable to any of these metrics, and results should be broadly similar. Therefore, for simplicity, we will only present results for one commonly used metric: PGA.

We adopted the Chiou and Youngs NGA-West2 GMPE^7^ because it is the smoothest function of magnitude among all the GMPEs developed from the NGA-West2 dataset of global earthquake observations. However, the results of this study should be robust to the choice of GMPE. The GMPE simply gives reasonable values for the median ground motion, and we assume the EEW system can perfectly predict that median ground motion. The accuracy of the EEW system is instead determined by the variability of the observed ground motion about the median, regardless of what that median is. This variability is considerable and is an important source of uncertainty in EEW^8,9^. Reducing uncertainty through incorporation of path- or site-specific geophysical information is an active area of research; however, current GMPEs typically have PGA standard deviations (including source, path, and site variability effects) of about a factor of two^10^. Thus:

1 $$\mathrm{log}\,PG{A}_{observed} \sim {\mathscr{N}}(\mathrm{log}\,GMPE({\boldsymbol{M}},{R}_{rup}),\,\mathrm{log}\,2)$$

where *PGA*_*observed*_ is the observed PGA, ***M*** is moment magnitude, *R*_*rup*_ is the distance between the rupture and site for which ground motion is being estimated, *GMPE(****M****, R*_*rup*_) represents the predicted median ground motion from the ground motion prediction equation (i.e., the ground motion expected by our ideal EEW system), and ${\mathscr{N}}$ represents the normal distribution. As shown below even our ideal EEW system, which perfectly knows the final magnitude and extent of the earthquake rupture and perfectly estimates the median expected ground motion, will produce missed alerts and false alerts because of this ground motion variability.

## Results

### Best possible accuracy for EEW

To determine the alerting accuracy for an ideal EEW system, we combine catalogs of earthquake magnitudes and locations with ground motion predictions, using a PGA GMPE^7^ to calculate median ground motions that are used to issue (or not issue) an alert for each user location. Because of PGA variability, however, the actual PGA at our user’s site might be quite different. This hypothetical ground motion is calculated here as the median value plus a random draw from the normal distribution given by eq. 1. It is the comparison between the perfect, median ground motion and the hypothetically observed ground motion that determines whether the decision to issue (or not issue) that user an EEW alert was made correctly.

If more observations of strong ground motion existed, we could compute statistics of EEW performance directly from observed records. However, even large databases of ground motion such as NGA-West2^11^ are simply not large enough to allow us to calculate robust statistics. So instead of calculating our statistics directly from strong motion records for a limited subset of well-recorded earthquakes, we have first created a much larger population of earthquakes distributed according to observed catalogs^11,12^ and then made our ground motion calculations from that larger population using GMPEs. However, these data should not be seen as synthetic. We are using the real distribution of observed ground motions in the NGA-West2 database^10^.

**Figure 2:**
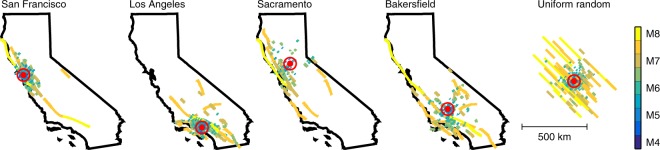
Selected ruptures contributing to each earthquake catalog. For San Francisco, Los Angeles, Sacramento, and Bakersfield, we plot 500 years of earthquake ruptures generated from UCERF3 probabilities. 500 sample ruptures from the uniform random catalog of ruptures are also plotted. Each line represents a single rupture, colored by earthquake magnitude. The bullseye marks the observation location. For visual simplicity, only ruptures that produce at least 2%*g* are shown. These ruptures are a subset of the full catalogs used in this study.

We use two types of simulated event catalogs. The first type is a 480,000-year duration catalog of **M**4-8 ruptures generated using probabilities from the third Uniform California Earthquake Rupture Forecast (UCERF3) with epidemic-type aftershock sequences^12^ (UCERF3-ETAS). We then calculate the median expected PGA from each of the 40,357,018 ruptures that occurred during this time period at each of four cities in California: San Francisco, Los Angeles, Sacramento, and Bakersfield (Fig. 2), yielding four different scenario earthquake catalogs. The first two are for large population centers located near major faults. At least for California, these urban areas have high seismicity rates, hazard, and risk. The other two are for cities further removed from major faults, with lower background seismicity rates and hazard.

**Figure 3:**
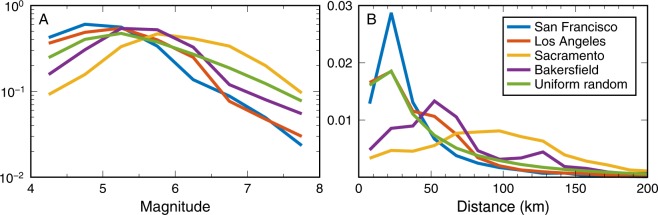
Normalized PDFs of magnitude and distance. PDFs of (**A**) magnitude and (**B**) rupture distance for 480,000 years of ruptures generated with UCERF3 probabilities that are observed to produce PGA > 2%*g* in San Francisco, Los Angeles, Sacramento, and Bakersfield. The PDFs for a catalog of earthquakes with uniform random location and constant strike are also shown. All PDFs are normalized to integrate to one, allowing better comparison between locations with different seismicity rates. (**A**) While the earthquake catalogs are generated using UCERF3 or Gutenberg-Richter probabilities, and thus start with approximately constant slope, we are only plotting the subset of earthquakes in the full catalog whose stochastic realizations of PGA exceed 2%*g* at a specified location. Thus distant small earthquakes are not included and the frequency of occurrence decreases at small magnitude. All locations produce approximately similar magnitude-frequency relationships. However, the distribution of rupture distances (**B**) is quite variable, with San Francisco and Bakersfield hazards being dominated by faults at specific distances, while Sacramento is mostly affected by background seismicity at all distances. Los Angeles, which is home to many faults following a dominant trend, is perhaps unsurprisingly similar to the uniform random catalog.

The other type of catalog we use is an assemblage of 10 million earthquakes with epicenters distributed uniform randomly across California. We have placed these ruptures in a regional stress field so that all ruptures have the same strike, roughly akin to the San Andreas fault system. For this simulation, we assume that earthquake magnitudes are Gutenberg-Richter distributed with slope one^13^, and range between **M**4 and **M**8. This produces a catalog whose frequency-magnitude and distance distributions are surprisingly similar to the UCERF3 catalogs, but with earthquake distance distributions that fall in between places like Sacramento (dominated by background seismicity) and San Francisco (dominated by the presence of several major faults) (Fig. 3). In fact, the distance distribution for the uniform random catalog is nearly identical to the UCERF3 catalog for Los Angeles, which suggests that the earthquake hazard in Los Angeles arises primarily from many sub-parallel faults, with magnitudes sampled from a Gutenberg-Richter distribution. However, the uniform random catalog is also completely generic and generated from a global NGA-West2 GMPE with a global model of ground motion variability, and thus should be applicable to any part of the world.

**Figure 4:**
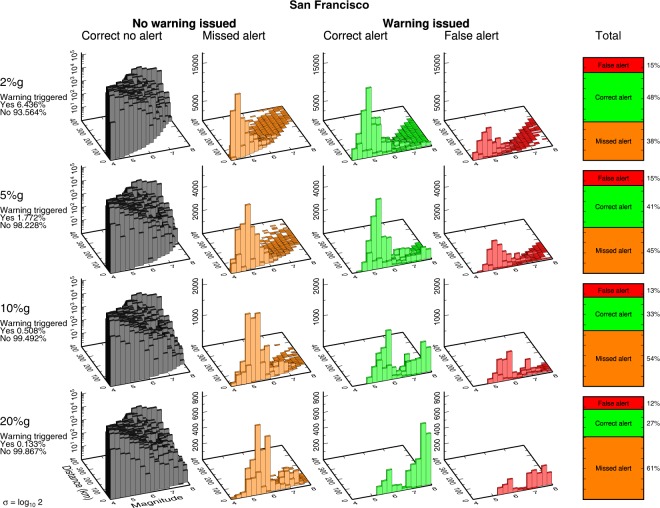
Deaggregated earthquake alert outcomes for San Francisco. Each earthquake in the UCERF3 catalog is categorized into (from left to right) a correct no alert, missed alert, correct alert, or false alert for 2%, 5%, 10%, or 20%*g* for a user located in San Francisco. Text on left hand side lists what percentage of the total number of events in the catalog does (Yes) or does not (No) trigger an alert. Because strong ground motions are more infrequent than weak shaking, fewer earthquakes trigger an alert for 5%*g* than for 2%*g*, and so on. On the right hand side, we ignore the correct no warning case and tally what percentage of the remaining events are classified as correct alerts, false alerts, or missed alerts. In almost all cases, the most common outcome a user will experience is a missed alert. This behavior becomes more pronounced at higher ground motion thresholds.

**Figure 5:**
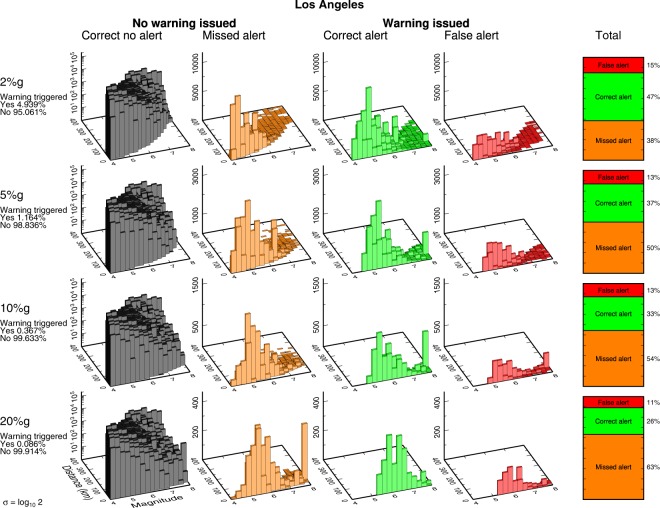
Deaggregated earthquake alert outcomes for Los Angeles. Same as Fig. 4 using Los Angeles as the target location.

**Figure 6:**
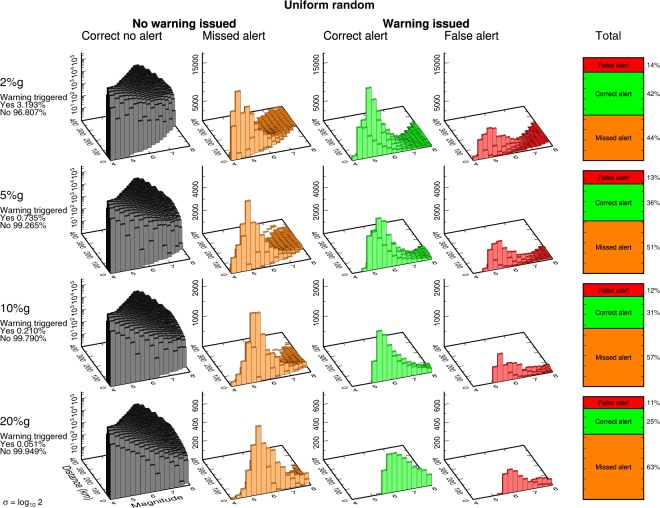
Deaggregated earthquake alert outcomes for randomly distributed ruptures. Same as Fig. 4 for the catalog of 10 million uniform random distributed ruptures.

We consider four levels of PGA for which a user might want to receive warning: 2%*g*, 5%*g*, 10%*g*, and 20%*g*. These thresholds correspond roughly to MMI levels IV (light shaking) through VII (very strong shaking), and are the same as those used in our companion study^5^ and also are roughly equivalent to PGV thresholds of 2 cm/s, 5 cm/s, 10 cm/s, and 20 cm/s. For each threshold and for each earthquake in the catalog, we assume that an alert is issued if the predicted median PGA at an imaginary user’s location in the city is greater than the threshold. Based on whether an alert is issued and whether our realization of observed PGA falls above or below the threshold, we assign each “event alert” one of the following four categories: correct alert, false alert, missed alert, and correct no alert (Fig. 1). We then deaggregate these assignments by magnitude and rupture distance for each alert threshold (2%*g*, 5%*g*, 10%*g*, and 20%*g*) using the UCERF3 catalogs for San Francisco, Los Angeles, Sacramento, and Bakersfield, and the uniform random catalog (Figs 4–6, S1–S2). The results for all five catalogs are similar in terms of the relative proportions of false, correct, and missed alerts. This comparison between the UCERF3 catalogs at four different locations with different local faults and different local site conditions as well as a generic random distribution of faults demonstrates that EEW accuracy is fairly insensitive to regional differences in the spatial distribution of faults or rupture probabilities.

The most common outcome the user experiences is the “correct no alert” case, reflecting myriad small or distant earthquakes that should not and do not produce significant ground motion at the user’s location. This result holds for each of our four cities and the generic uniform random catalog. (Note that the deaggregated frequency of occurrence of this case is shown on log scale in Figs 4–6, S1–S2, because it is so plentiful.) The “correct no alert” population is also arbitrarily large: if we expanded our earthquake catalog to include smaller and more distant earthquakes, we would grow the number of correct no alerts exponentially while the number of correct, false, and missed alerts would remain constant, since none of these small or remote earthquakes would produce shaking or trigger alerts. Because the correct no alert case is both arbitrary and not of any practical significance, we will mostly ignore it.

For the correct, false, and missed alerts, we see that the most common outcome in almost every case is missed alerts. While the relative proportions of missed and correct alerts are nearly identical at the 2%g threshold, missed alerts increase rapidly when the ground motion threshold is increased until missed alerts are overwhelmingly dominant at high ground motion thresholds (Fig. 6). For example, there are 63% missed alerts and only 25% correct alerts for the uniform random catalog at the 20%*g* threshold. Scaling relationships point to the cause of the abundance of missed alerts. The frequency of occurrence of earthquakes scales strongly with magnitude, typically log N ∼ −1 M, i.e., the familiar Gutenberg-Richter relationship^13^. Ground motion scales more weakly with magnitude, log PGA ∼ 0.3 M^14^. Thus, because of the Gutenberg-Richter distribution, there are always many earthquakes with expected ground motion just below the threshold, whatever the choice of threshold. Nominally, these earthquakes should lead to the “correct no alert” case. However, given ground motion variability, it is very easy for some of this population to cross from “correct no alert” to “missed alert”.

As an example, consider the 20%*g* threshold: our ideal EEW system always correctly knows the magnitude and rupture extent and correctly estimates the median expected ground motion. Large (and close) earthquakes that are expected to produce ground motion exceeding 20%*g* – and thus trigger an alert – result in correct alerts much more often than they result in false alerts (Fig. 1A). Similarly, small (or distant) earthquakes almost always result in the trivial “correct no alert” case and rarely result in missed alerts (Fig. 1B). Large and/or close earthquakes expected to exceed 20%*g* are very rare: only 0.051% of all earthquakes in the uniform random catalog are expected to produce that level of shaking at the user location. Thus the total population of correct and false alerts for 20%*g* PGA is small. And while only a tiny fraction of the small and/or more distant earthquakes will result in unexpectedly large ground motion exceeding 20%*g*, it is a tiny fraction of an enormous population of small events (due to Gutenberg-Richter magnitude statistics). Thus the absolute number of missed alerts is large, and the most common outcome a user will experience is a missed alert.

### Effects of alerting strategy on alert accuracy

**Figure 7:**
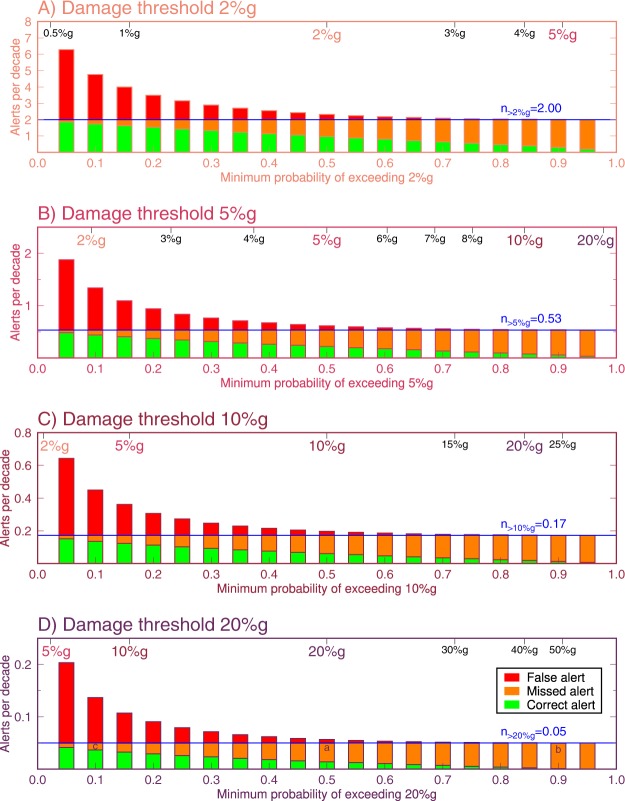
Alert outcomes as a function of different alerting thresholds. For our four damage thresholds of (from top to bottom) 2%*g*, 5%*g*, 10%*g*, and 20%*g* in Fig. 6, we categorize each earthquake into correct, missed, and false alerts (following the definitions in Fig. 1C) for different choices of alerting threshold shown on the top *x*-axis. In Figs 4–6, S1–S2, we assumed the alerting threshold was equal to the damage threshold, or equivalently that the probability of exceeding the damage threshold was 0.5. In this figure, we have relaxed that assumption, with the bottom *x*-scale defining the alerting threshold in terms of the probability of exceeding the damage threshold. The top *x*-scale defines the alert threshold as the equivalent minimum expected median ground motion that would trigger an alert. We plot the histograms in units of how many occurrences of each outcome is expected per decade given typical California seismicity rates. The blue line shows the number of times per decade that the observed ground motion exceeded the damage threshold; these events can only result in missed or caught alerts, although the relative proportion of each changes with the alerting threshold. If the user only wishes to take action when there is a high probability that the ground motion will exceed the damage threshold (right-most bars in each subplot), then there will be few false alerts but most damaging earthquakes will be missed. If instead, the user wishes to make sure that an alert is always received before damaging ground motion is experienced, the alerting probability can be set low (left-most bars in each subplot). This will result in few missed alerts at the cost of many false alerts since most of the earthquakes for which the user takes action will not be damaging. Letters a, b, and c in (D) indicate examples discussed in text.

In the previous section, we have assumed that the ideal EEW system will issue an alert if the expected ground motion, i.e., the median ground motion output by the GMPE, is higher than a user’s damage threshold. This is equivalent to saying that an alert will be issued when the probability of exceeding the target ground motion threshold is at least 0.5, or 50%. In Fig. 7, we generalize our alerting criteria and define two thresholds instead of one: a damage threshold at which some injury, personal anxiety, or material loss occurs; and an alerting threshold at which the system decides to send an alert (Fig. 1C). We examine the performance effects of setting these two thresholds to be different from one another.

Again, up to now, we have set the alerting and damage thresholds to be equal – an alert would be issued when there is at least 0.5 probability that ground motion will exceed the damage threshold at a user’s location because the GMPE predicts median ground motion exceeding the damage threshold. For a user who suffers damage when ground motion exceeds 20%*g*, for example, with the uniform random catalog of 10 million earthquakes, the user would receive a correct alert for only 28% of damaging earthquakes. At that same alerting threshold, 72% of damaging earthquakes would be missed and 31% of alerts received would be false, i.e., ground motion did not exceed 20%*g* (shown by letter “a” in Fig. 7D, based on outcomes in Fig. 6D).

Perhaps a user would be dissatisfied with the percentage of false alerts that resulted in taking action when no damage was expected, as could be the case if the cost of taking action is very expensive. Instead the user may decide to only take action if there were at least 0.9 probability of exceeding 20%*g*, which is equivalent to taking action when the median expected ground motion is >48.6%*g* (“b” in Fig. 7D). In that case, the number of false alerts would be decreased significantly to just 6% of alerts. However, for this choice of alerting threshold, 98% of damaging earthquakes would be missed.

Alternatively, a user may prefer that no damaging earthquake was missed, and decide to take action when there was just 0.1 probability of expected ground motion exceeding 20%*g*, equivalent to taking action when the median expected ground motion is just 8.2%*g* (“c” in Fig. 7D). In this scenario, the user would receive a correct alert for 73% of damaging earthquakes, although 27% of damaging earthquakes will still be missed. But the cost of this more aggressive alerting strategy is that now 70% of alerts received will be false: unnecessary action will be taken for many small earthquakes that never exceed 20%*g* ground motion.

This demonstrates that the performance of an EEW system is highly sensitive to the choice of alerting threshold relative to a user’s damage threshold. But further, it demonstrates that there is a need to determine the optimal alerting strategy for each user. The paramount question is: are false alerts better or worse than missed alerts? For the former the alerting threshold should be set low; for the latter, the alerting threshold should be set high.

The basic concept of EEW is that, with sufficient warning, there is an action that can be taken to prevent or mitigate damage. There is some cost associated with that action, but the cost of taking action must be less than the cost associated with the unmitigated damage or EEW would not be useful for that application. Thus missed alerts must always be more costly than false alerts, and for EEW to be effective all EEW users must be, at least to some extent, false-alert-tolerant. The only question is: how false-alert-tolerant is the user? If the user is very false-alert-tolerant, then the alerting threshold should be set very low to minimize the number of missed alerts even if that results in many false alerts. Users who are only slightly false-alert-tolerant may achieve optimal performance with higher alerting thresholds. However, we anticipate that even these users will be best served by an alert threshold that is significantly below the damage threshold because, if the alerting threshold is set to the damage threshold, the most common outcome will be missed alerts (Figs 4–6 and S1–S2).

### Optimal alerting strategy

To identify the best alerting strategy, we find the alerting threshold that maximizes the utility of EEW to the user. We do this by minimizing the costs associated with earthquakes. This is hardly a formal benefit-cost analysis, simply the creation of a cost function that can be optimized to find the best EEW alerting strategy. Let us define *C*_*damage*_ as the cost of the damage that can be prevented if the user receives an alert and takes mitigation action, and *C*_*action*_ as the cost of that action. Then, with an operating EEW system, the net cost to the user is:

2 $${C}_{EEW}={C}_{action}{N}_{correct}+{C}_{damage}{N}_{miss}+{C}_{action}{N}_{false}$$

where *N*_*correct*_, *N*_*miss*_, and *N*_*false*_ are the number of correct, missed, and false alerts, respectively. The three terms in eq. 2 can be identified, from left to right, as the costs associated with taking preventative action during correct earthquake alerts, the preventable losses suffered due to missed alerts, and the cost associated with taking unnecessary action in response to false alerts.

If EEW did not exist, then the cost associated with preventable damage from these same earthquakes would be:

3 $${C}_{withoutEEW}={C}_{damage}({N}_{correct}+{N}_{miss})$$

This is simply the preventable cost associated with all damaging earthquakes, which is the sum of the number of earthquakes resulting in either correct or missed alerts in an EEW context (*N*_*damage*_ = *N*_*correct*_ + *N*_*miss*_).

Let *r* = *C*_*damage*_/*C*_*action*_; this ratio defines how false-alert-tolerant the user is. EEW is only useful to a given user if *r* >1, that is, that the damage you are trying to prevent is worse than the action necessary to prevent that damage. Large *r* implies the cost of action is low compared to the cost of damage, such as protective “drop-cover-hold on” actions to avoid falling objects; small values of *r* imply high cost of action compared to the cost of damage, such as a nuclear scram (an emergency reactor shutdown that can be used as earthquake response action)^15,16^.

Let us further define a missed alert rate, *m* = *N*_*miss*_/*N*_*correct*_, and a false alert rate, *f* = *N*_*false*_/*N*_*correct*_. We can then combine eqs 2 and 3 to make a statistic that we call the normalized cost reduction due to EEW:

4 $$CR=\frac{{C}_{withoutEEW}-{C}_{EEW}}{{C}_{withoutEEW}}\times 100 \% =\frac{1-\frac{f+1}{r}}{m+1}\times 100 \% $$

Equation 4 non-dimensionalizes and normalizes the cost function to be independent of the actual costs. *CR* = 100% means that, due to the usefulness of EEW, all earthquake losses have been prevented. *CR* = 0% means that EEW does not prevent any earthquake losses. And *CR* < 0 implies that the number of false alerts is so great that EEW results in a net loss to the user. Note that eq. 4 is independent of the number of earthquakes or seismicity rate – it simply depends on the relative frequency of correct, missed, and false alerts. Further, it is not necessary to know the actual costs to the user, just the ratio between the costs of the damage that could be prevented and the cost of taking action. This, in turn, can be replaced with a metric of how false-alert-tolerant the user is.

**Figure 8:**
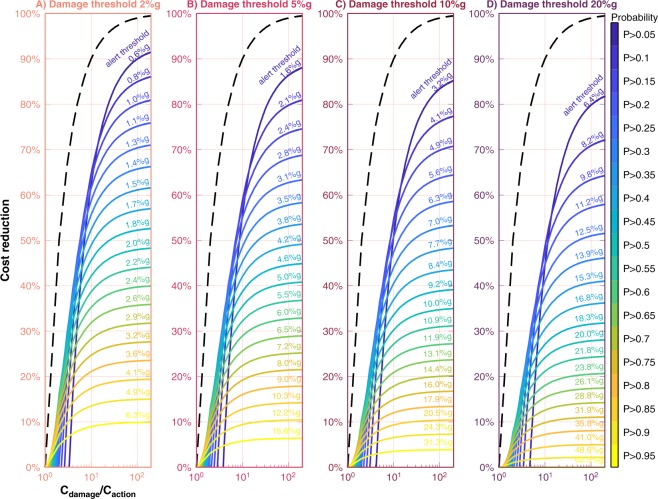
Potential cost reduction. For our four damage thresholds of (from left to right) 2%*g*, 5%*g*, 10%*g*, and 20%*g*, we compute the cost reduction (eq. 4) as a function the ratio *r* = *C*_*damage*_/*C*_*action*_, the ratio of the cost of the damage that could be prevented to the cost of taking action to prevent that damage. This can be viewed as false alert tolerance. Users for whom *r* is large (the right end of each *x*-axis) are very false-alert-tolerant. Users for whom *r* approaches one (left end of each *x*-axis) are only slightly false-alert-tolerant. Each colored line corresponds to a different choice of alerting threshold, which can be specified by probability of exceeding the damage threshold (probabilities shown on color scale) or equivalently by the minimum median expected ground motion corresponding to that probability (shown by annotations next to each curve). Each line is calculated from one set of stacked histograms in Fig. 7, and the color scale and text annotations correspond to the bottom and top *x*-axes, respectively, in Fig. 7. The black dashed line is the maximum possible cost reduction for an EEW system that has zero missed alerts and zero false alerts.

As we adjust the alerting threshold (e.g., move along the upper *x*-axis of Fig. 7), we change the number of correct, missed, and false alerts, altering *f* and *m* in eq. 4. This in turn changes the performance of the EEW system as seen by that user, and we can quantify the effectiveness of the EEW system using eq. 4. For the outcomes of each alerting threshold (Fig. 7), we compute the cost reduction as a function of *r* = *C*_*damage*_/*C*_*action*_ (Fig. 8). Then, it is clear that more benefit (larger *CR*) can be derived from EEW for users that are very false alert tolerant (*r* large), and that the maximum benefit is achieved by choosing a very low alerting threshold (relative to the damage threshold) in order to minimize the number of missed alerts despite greatly increasing the number of false alerts (Fig. 8). In fact, this is generally true for all types of natural hazards warning not just EEW^17^.

For example, consider a highly false alert tolerant user for whom *r* = 100 and who experiences damage if ground motion exceeds 20%*g* (right side of Fig. 8D). The seemingly most straightforward strategy would be to take action every time that the EEW system calculates that expected median ground motion is greater than 20%*g* (i.e., that the probability of exceeding 20%*g* is at least 0.5). However, this results in many missed alerts (Figs 6 and 7), and the user would only see a cost reduction of 28% (Fig. 8D). Instead, if that user took action if there were a probability of just 0.05 that the ground motion would exceed 20%*g* (equivalent to taking action when the median expected ground motion is only 6.4%*g*), the user could achieve 80% cost reduction even though, in this scenario, 79% of all alerts received would turn out to be false alerts.

Note that *CR* = 100% is not possible even for a perfect EEW system that has zero missed events and zero false events. To illustrate this, imagine a printer whose printing presses will jam under strong ground motion, causing $1000 in damage. Given a warning, the printer could shut down the presses in an orderly fashion and restart them after the ground motion has ceased, preventing the jam but costing $200 in lost productivity. While EEW provides this user with significant cost reduction, the printer still loses $200 every time there is an earthquake. For this fictional printer, *r* = $1000/$200 = 5. Thus the maximum cost reduction the printer could enjoy, even if the EEW system never produced a false or missed alert, is an 80% cost reduction (per eq. 4 with *f* = *m* = 0). This maximum possible cost reduction for a perfect system without false or missed alerts is shown by the dashed black line in Fig. 8, leading us to the following conclusion: EEW produces little cost reduction for users for whom the cost of taking action is almost as expensive as the cost of the damage they are trying to prevent. (Again, “damage” can be anything including infrastructure harm, human injuries, or emotional distress). EEW has its greatest potential for users for whom *r* is large; or rather, EEW has its greatest potential for users who are false-alert-tolerant. And for those users, optimal performance is achieved by taking action for even small (or distant) earthquakes that are unlikely to cause damage (Figs 8, S3).

### Effects of damage uncertainty

Up to now, we have assumed that there is a damage threshold: ground motion above that threshold will cause damage, while ground motion below that threshold will not be damaging (and any associated earthquake alert will thus be a false alert that triggered unnecessary action). In real-world systems, the relationship between ground motion and damage is generally more complicated and is described probabilistically by a fragility curve^18^ (Fig. S4). This curve describes the relative likelihood that any particular level of ground motion will result in damage. It is straightforward to apply the analysis in this paper to a probabilistic damage model by simulating damage outcomes and assessing the “correct” vs. “false” alert and “missed” vs. “correct no alert” classifications relative to whether or not damage occurred rather than whether or not a ground motion threshold was exceeded (Figs S5–S6). All of this simply makes it more uncertain as to whether the user should take protective action for any particular earthquake, thereby diminishing the practical utility of EEW alerts. However, the conclusions are the same: EEW is most useful for users who are false-alert-tolerant, and the optimal alerting strategy for these users is to take action even when the predicted shaking is weak and unlikely to cause damage.

## Discussion

Intuitively, one might expect that EEW should alert users for ground motion expected to exceed some level that causes damage: if some piece of factory equipment will be damaged by accelerations greater than 50%*g*, then send an alert to shut down that equipment if the expected ground motion is equal to or greater than 50%*g*; otherwise continue operations. However, this straightforward approach would yield surprisingly poor alert performance. There are two reasons for this. First, most of the time that ground motion exceeds a specified threshold it is caused by a small earthquake producing unexpectedly strong ground motion. In these cases, the EEW system would not issue an alert because small earthquakes are not expected to produce damaging shaking. So receiving alerts for only earthquakes with expected median ground motions above some level that could cause damage would result in the user not being alerted for the majority of earthquakes that cause potentially damaging ground motion for that user (Figs 4–6, S1–S2). This behavior is the result of the combination of ground motion variability with the fact that small earthquakes are much more common than large earthquakes. Second, this effect is amplified by the fact that missed alerts must be more costly than false alerts. If the cost of taking action to prevent some outcome is more expensive than the cost of that outcome, then EEW has no benefit. Thus optimal alerting performance is obtained when the alerting threshold is set to be significantly lower than the user’s damage threshold; but for any individual alert, there is only a small probability that damaging ground motion will occur. This approach minimizes the number of missed alerts and maximizes users’ gains even though it also greatly increases the number of false alerts.

Currently, there is no robust research to indicate what individual members of the public might desire or expect from EEW. There may be some societal benefits of EEW that have nothing to do with successfully warning people. For example, people may enjoy receiving real-time earthquake information even if it arrives after the dangerous shaking has already happened, or appreciate being given earthquake information even if they do not understand it^19,20^. But we cannot assess these possible societal impacts at this time. What we can do is assess how often an EEW system is able to realize the physical and emotional benefit (to infrastructure and humans) of being successfully warned to take protective action before dangerous ground motion arrives at a location: the direct effectiveness and accuracy of an EEW system.

The amount of benefit a user can achieve following this approach depends on the relative cost of the damage that user is averting by taking action triggered by an EEW alert. If the cost of taking action is almost as expensive as the damage the action is meant to avoid, then EEW can provide little benefit even if it never produced missed or false alerts (black dashed line in Fig. 8). And EEW alerts that have large uncertainty in expected ground motion are likely to have little utility to users whose mitigating actions are very expensive. For example, EEW may not be a good earthquake risk mitigation tool for nuclear power plants because an emergency shutdown costs more than $250 million not including other costs such as the resulting decrease in the lifetime of the reactor^16^.

The false-alert tolerant user, however, is the more interesting case. Almost any able-bodied person could take “drop-cover-hold on” action at very low ground-motion thresholds to mitigate damage or injury in the case of stronger ground motion. The act of “drop-cover-hold on” is essentially free and only takes a few seconds, resulting in very little loss of productivity. Yet the resulting benefit of being under a desk, say, rather than getting hit by a toppling bookshelf or falling light fixture is worth much more than the loss of productivity. That is, the cost of damage is much more than the cost of action. The many false alerts that this general user would experience under this alerting strategy might even act as preparedness drills^19^.

The results we report here are specific to EEW systems that use source information (the location and magnitude of an earthquake) to calculate the expected ground motion using a GMPE. This is the most popular type of EEW in use today. (All of the algorithms comprising the United States’ ShakeAlert system and all but one of Japan’s JMA EEW system’s algorithms are of this type^1,2^). However, ground motion estimation rather than source parameter determination is the ultimate goal of an EEW system, and magnitude (even when it is exactly estimated) is not a sufficient source parameter for accurately predicting ground motion.

Fortunately, other EEW methods are under development that use observations of ground motion to directly calculate expected ground motion without inferring source parameters or implementing a GMPE^21–23^. Expected ground motion variability in this method is potentially lower as the effects of inter-event variability are implicitly modeled, whereas it is a large part of the factor of two uncertainty in GMPE predictions (eq. 1). Specifically, the total variability could be reduced by ~20% to just the within-event variability^24^. A similar effect could be possible for source-parameter-based EEW if, during the alert generation process, observed ground motions were used to update the event term of the GMPE (a measure of how energetic the earthquake is) to match the ongoing earthquake. Additional improvements in alert accuracy could be obtained by the use of path- and site-specific GMPEs in addition to event-specific corrections^25^ (Figs S7–S9). On the other hand, users who wish to predict shaking within a building will have to deal with additional uncertainty in modeling the structural response to the ground motion at its base.

In this study, we have considered the accuracy of an ideal EEW system that has instantaneous knowledge of the final earthquake source (magnitude and rupture extent) and uses a GMPE that always perfectly calculates the median expected ground motion. Given realistic estimates for ground motion variability, we expect that even such an ideal system would provide incorrect alerts the majority of the time. While ground motion variability rarely results in false alerts (alerts for earthquakes that do not produce damaging ground motion), the combination of ground motion variability and Gutenberg-Richter statistics results in missed alerts for the majority of damaging earthquakes. In addition to these shortcomings, real-world EEW systems can suffer missed and false alerts due to errors in the inferred source parameters or the latency required to observe enough moment release to trigger an alert (the subject of our previous paper^5^), as well as missed alerts when the system fails to detect a damaging earthquake, or false alerts when it is tricked into thinking that an earthquake is occurring when one is not^26^. Source parameter errors further degrade the accuracy of EEW ground motion predictions. For example, given a magnitude 6 earthquake at 20 km distance and using our reference ground motion model^7^, a ±0.5 magnitude unit error or ±5 km distance error could result in varying PGA predictions of <4%*g* to >10%*g* and <6%*g* to >10%*g*, respectively, further increasing the chance the system will miss an alert or issue a false alert.

Nevertheless, even given these inaccuracies, EEW could still mitigate a large percentage of preventable earthquake damage for false-alert-tolerant users. This is accomplished by taking action when the expected ground motion is far below the level that can cause the user damage. Fortuitously, in our study of EEW timeliness^5^, we found that EEW alerts were more likely to be timely for low ground motion thresholds than for high ground motion thresholds. Thus, there is a simple path to ensure that users have the most effective EEW possible: alert for low levels of ground motion.

## Methods

For the UCERF3 examples, we used a 480,000-year duration catalog of 4 ≤ **M** ≤ 8 ruptures generated with UCERF3 probabilities^12^. The predicted ground motions for these ruptures were calculated using an NGA-West2 PGA GMPE^7^ for a global default region assuming the site is not on a hanging wall and that there is no directivity, and taking the basin depth term to be the median (default) for the model. The depth to the top of the rupture is taken as the median of the NGA-West2 database: 7 km for magnitudes less than 5, and then decreasing smoothly to 0 at **M** ~ 7.2. Vs30 values for each of the four locations studied are topographic proxy values^27^. Specifically, the assumed Vs30 values for San Francisco, Los Angeles, Sacramento, and Bakersfield, were 311.1195, 368.3053, 322.8545, and 268.4550 m/s. For each site, we then retained all earthquakes for which the predicted ground motion was at least 0.1%*g*.

The predicted ground motions for the generic uniform random distribution of earthquakes are calculated similarly except the Vs30 value is set to a generic rock site condition of Vs30 = 760 m/s. The catalog was created by randomly generating earthquake locations with uniform density within a 400-km-radius circle and magnitudes whose frequencies are Gutenberg-Richter distributed, 4 ≤ **M** ≤ 8, with a slope of one. Each earthquake was assumed to have constant strike, and its rupture length was specified by its magnitude using magnitude-log area scaling^28^ with a rupture width of 15 km. The first 10 million earthquakes whose predicted ground motion at the user’s location was at least 0.1%*g* were retained.

For both the UCERF3 and generic uniform random catalog, observed ground motions were taken to be the median expected ground motion plus a random draw of Gaussian noise with a specified standard deviation. A standard deviation corresponding to a factor of two in uncertainty was used  throughout except for Figs S7–S9^29–31^. See eq. 1 for details.

## Supplementary information

Supplementary Information

## Data Availability

All data generated or analysed during this study are included in this published article (and its supplementary information files) or are available from the corresponding author on reasonable request.
