# Supplementary material for: The Limits of Earthquake Early Warning Accuracy and Best Alerting Strategy
Source: Sci Rep. 2019 Feb 21;9:2478. doi: 10.1038/s41598-019-39384-y (PMC6385233; doi:10.1038/s41598-019-39384-y)
Supplement: Supplementary file 1 — Supplementary Information [file 41598_2019_39384_MOESM1_ESM.pdf]

# Supplementary information for: The Limits of Earthquake Early Warning Accuracy and Best Alerting Strategy

Sarah E. Minson[1,*], Annemarie S. Baltay[1], Elizabeth S. Cochran[2], Thomas C. Hanks[1], Morgan T. Page[2], Sara K. McBride[1], Kevin R. Milner[3], Men-Andrin Meier[4]

[1]U.S. Geological Survey, Menlo Park, California, 94025, USA
[2]U.S. Geological Survey, Pasadena, California, 91106, USA
[3]University of Southern California, Los Angeles, California, 90089, USA
[4]California Institute of Technology, Pasadena, California, 91125, USA
[*]sminson@usgs.gov

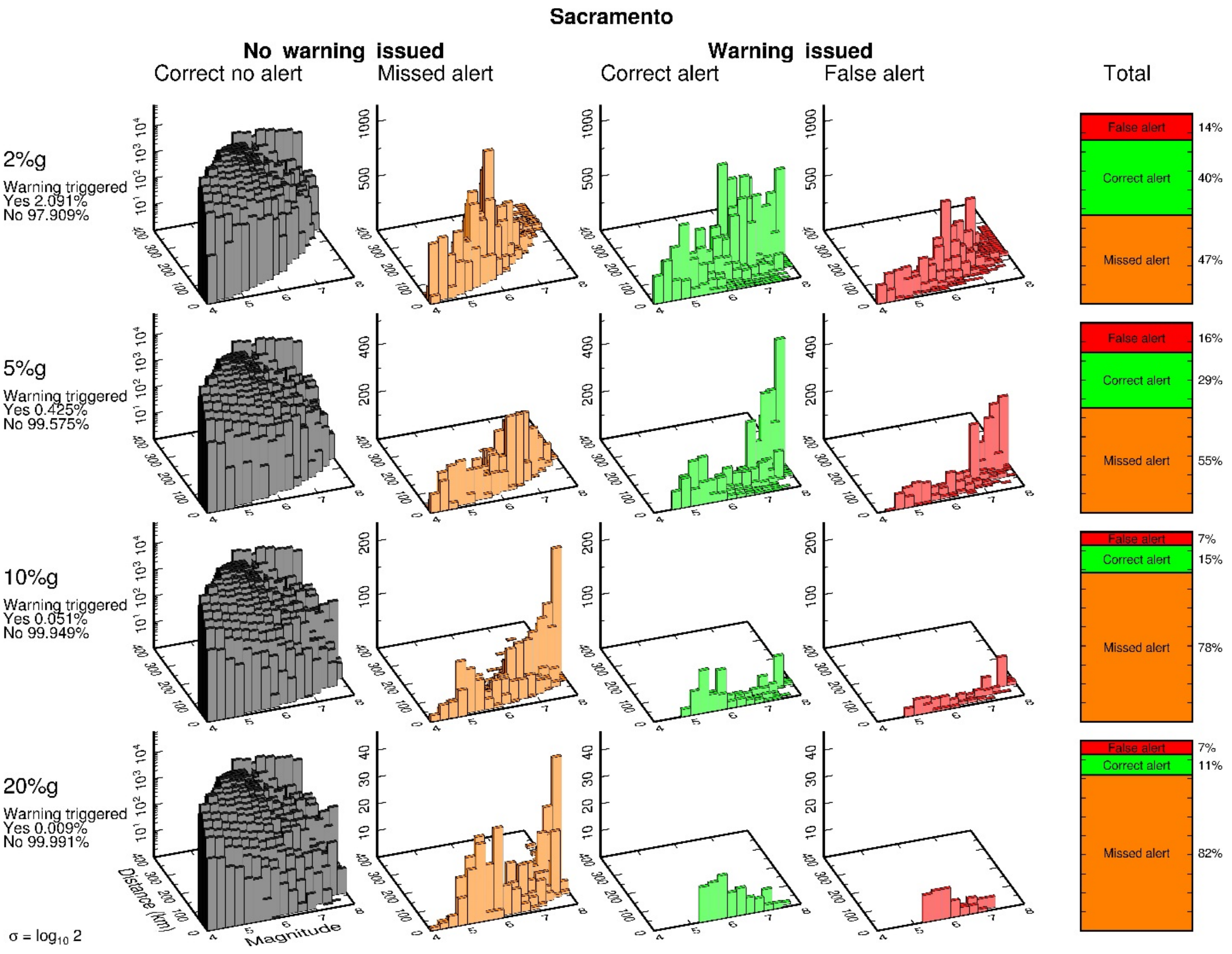


**Fig. S1. Deaggregated earthquake alert outcomes for Sacramento.**
Same as Fig. 4 for the UCERF3 catalog using Sacramento as the target location.

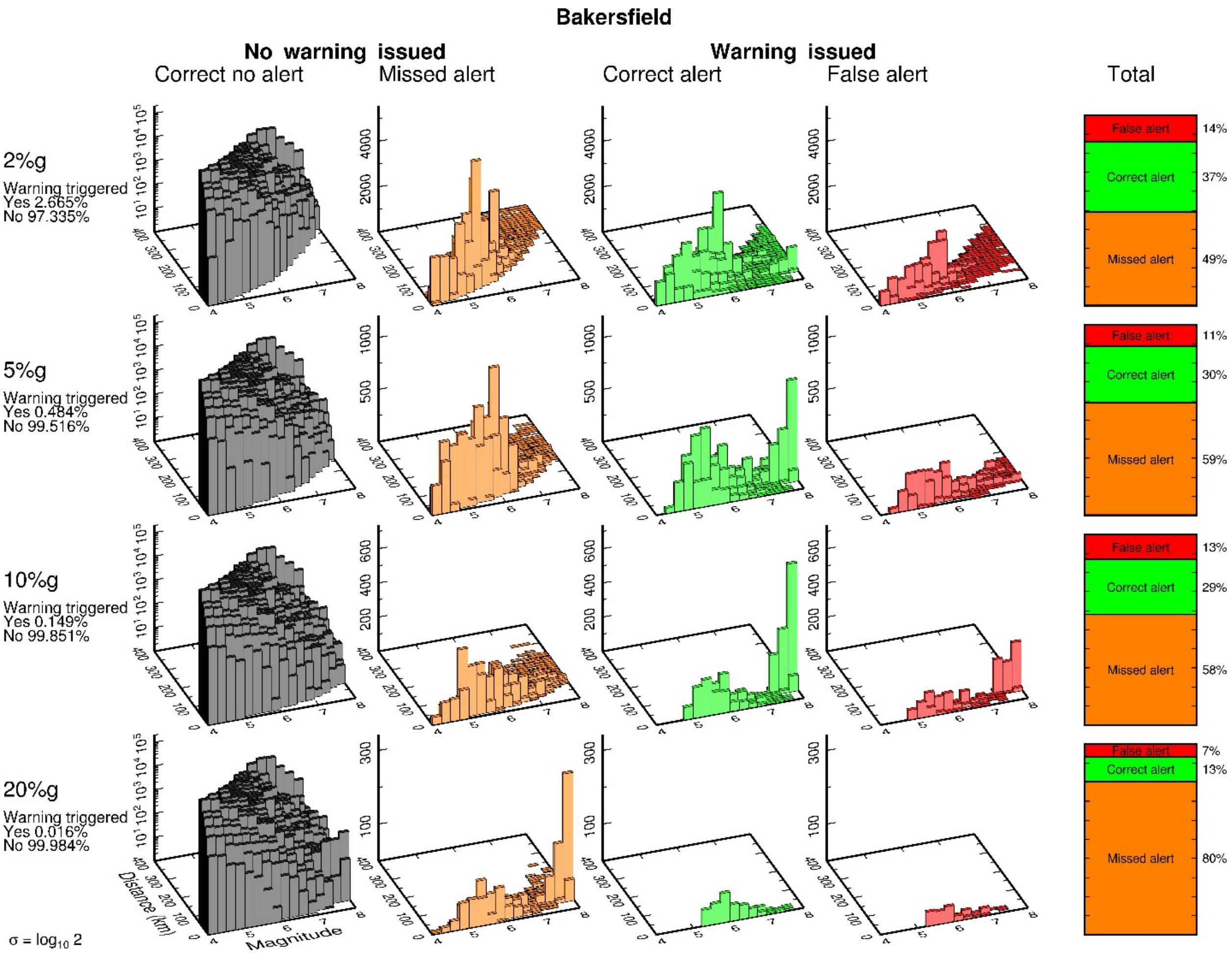


**Fig. S2. Deaggregated earthquake alert outcomes for Bakersfield.**
Same as Fig. 4 for the UCERF3 catalog using Bakersfield as the target location**.**

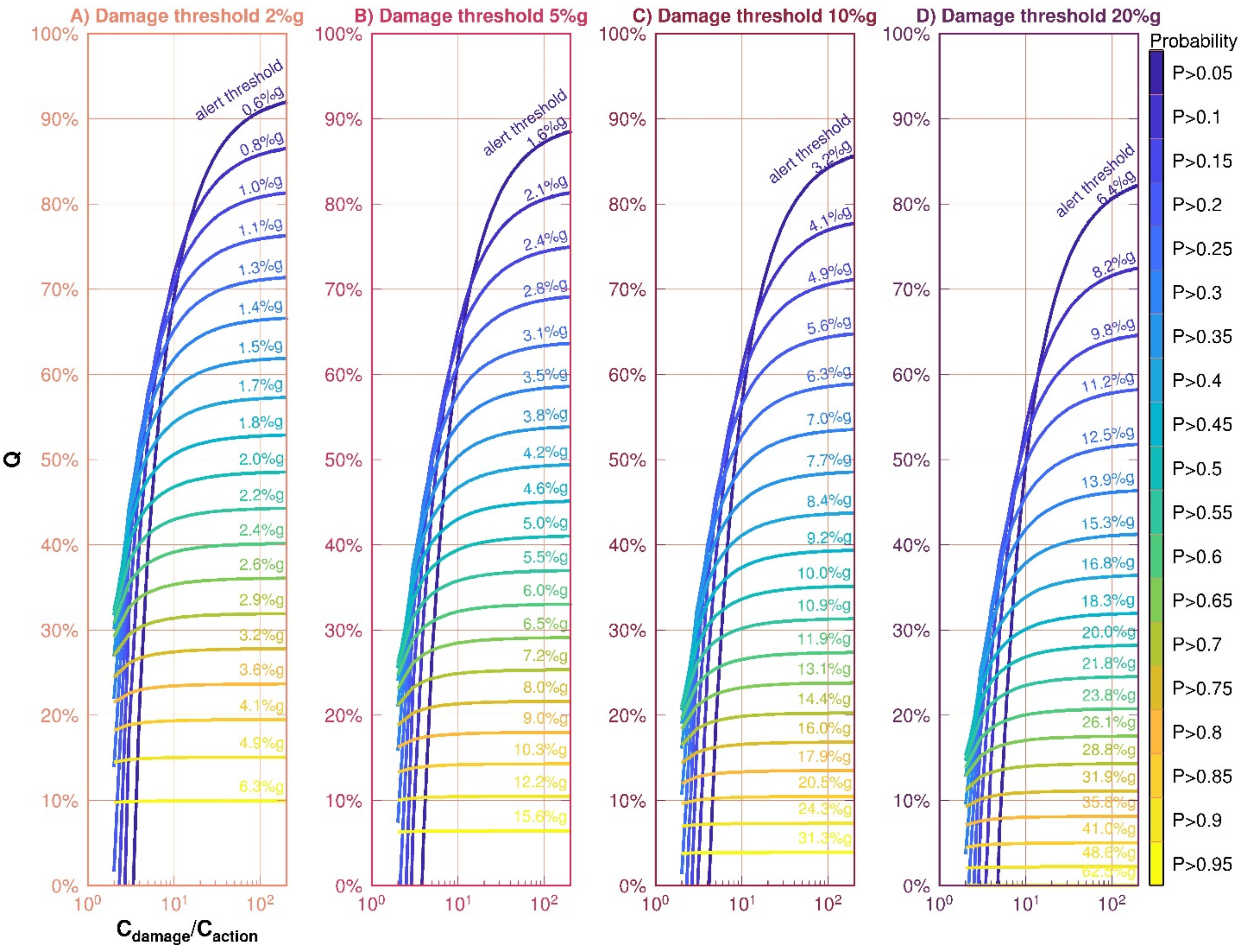


**Fig. S3. Percent of potential cost reduction realized.**

Same as Fig. 8, but instead of evaluating system performance by the total cost reduction, we compute the percentage of the maximum possible cost reduction that has been achieved. We define this quantity, $Q$, as the ratio of the observed value of eq. 4 to the value of eq. 4 with $f$=$m$=0:

$$Q = \frac{C_{without\ EEW} - C_{EEW}}{C_{without\ EEW} - C_{perfect\ EEW}} \times 100\% = \frac{1 - \frac{f}{r-1}}{m+1} \times 100\% \qquad (5)$$

Where $C_{perfect\ EEW} = C_{action}N_{damage}$ is the cost associated with a perfect EEW system that never has missed or false alerts. $Q$=100% implies that the EEW system has realized the theoretical maximum performance (black dashed line in Fig. 8). $Q$=0% means that the system provides no cost savings to users. And $Q$<0 means that there are so many false alarms that EEW is a net loss to users.

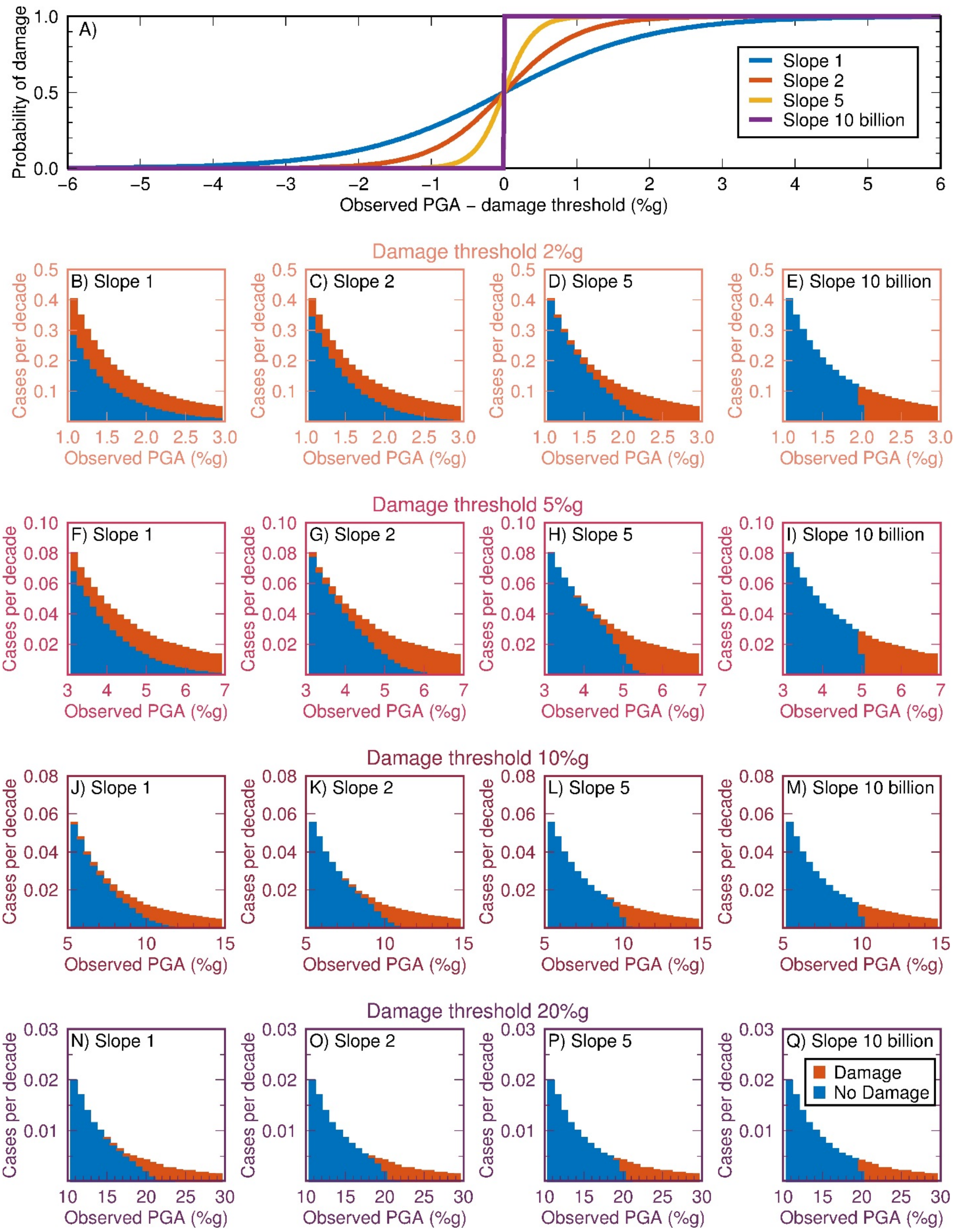

A)
Probability of damage
Observed PGA – damage threshold (%g)
Slope 1
Slope 2
Slope 5
Slope 10 billion
Damage threshold 2%g
B) Slope 1
C) Slope 2
D) Slope 5
E) Slope 10 billion
Damage threshold 5%g
F) Slope 1
G) Slope 2
H) Slope 5
I) Slope 10 billion
Damage threshold 10%g
J) Slope 1
K) Slope 2
L) Slope 5
M) Slope 10 billion
Damage threshold 20%g
N) Slope 1
O) Slope 2
P) Slope 5
Q) Slope 10 billion
Damage
No Damage
Cases per decade
Observed PGA (%g)

**Fig. S4. Sample fragility curves and resulting frequency of damage occurrence for different observed ground motions.**

**(A)** Comparison of sigmoidal fragility curves with different slopes. Each curve describes the probability that damage will occur at a given ground motion level. Specifically, we use the logistic function, $P = \frac{1}{1+e^{-k*(PGA\ observed - damage\ threshold)}}$, where *P* is the probably of damage occurring as a function of the observed PGA and some nominal damage threshold, and *k* is the specified slope. Shallow slopes imply that it is very uncertain as to which ground motion levels will result in damage. Steep curves imply that ground motion exceeding some threshold will almost certainly result in damage. The example with a slope of 10 billion should be effectively identical to the examples computed in the main text assuming that damage always occurs when ground motion exceeds some critical threshold. **(B-E)** Realizations of which observed PGA values resulted in damage and which did not using the uniform random distribution and fragility curves with slopes 1, 2, 5, and 10,000,000,000, respectively, centered around a critical damage threshold of 2%*g*. Stacked histograms show the number of earthquakes that resulted in damage or no damage, respectively, in units of number of occurrences per decade given typical California seismicity rates. (**F-I)** Same as (B-E) with fragility curves centered on 5%*g*. **(J-M)** Fragility curves centered on 10%*g*. **(N-Q)** Fragility curves centered on 20%*g*.

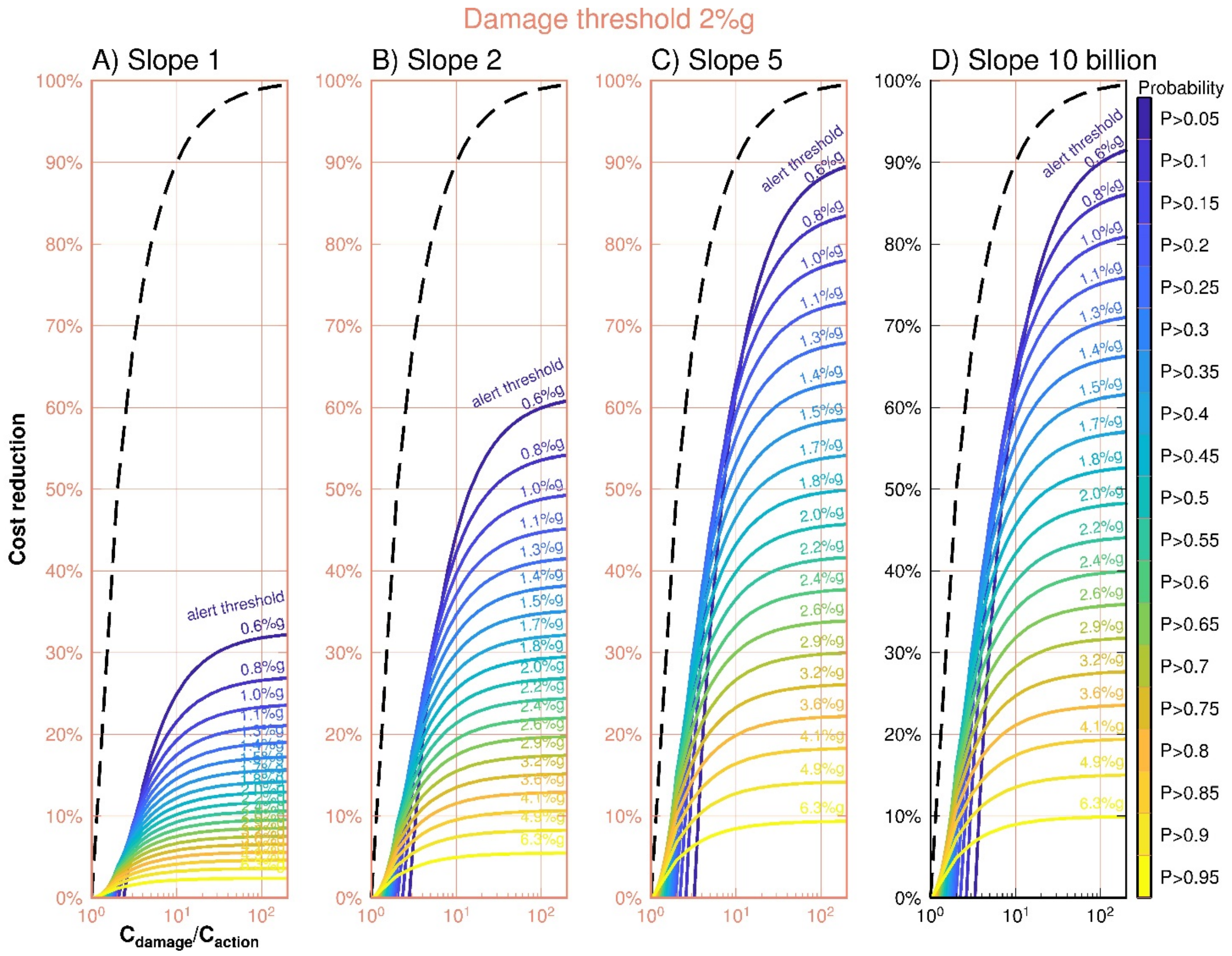


**Fig. S5. Potential cost reduction for different fragility curves centered about 2%*g*.**
Same as Fig. 8(A) for the fragility curves and damage realizations shown in Fig. S4(B-E). (i.e., the fragility curves are centered about a damage threshold of 2%*g*.) The alerts are categorized based on whether or not damage occurred: an alert for an earthquake that resulted in damage is classified as "correct" and an alert for an earthquake that did not result in damage is classified as "false" regardless of how large the observed ground motion is. (D) and Fig. 8(A) should be identical since a sigmoid function with a slope of 10 billion is effectively a step function. Note that the potential cost reduction increases as the slope steepens. This is because, the shallower the fragility curve, the more uncertainty in whether a particular ground motion will produce damage, further degrading the potential usefulness of EEW on top of the loss of accuracy due to ground motion variability.

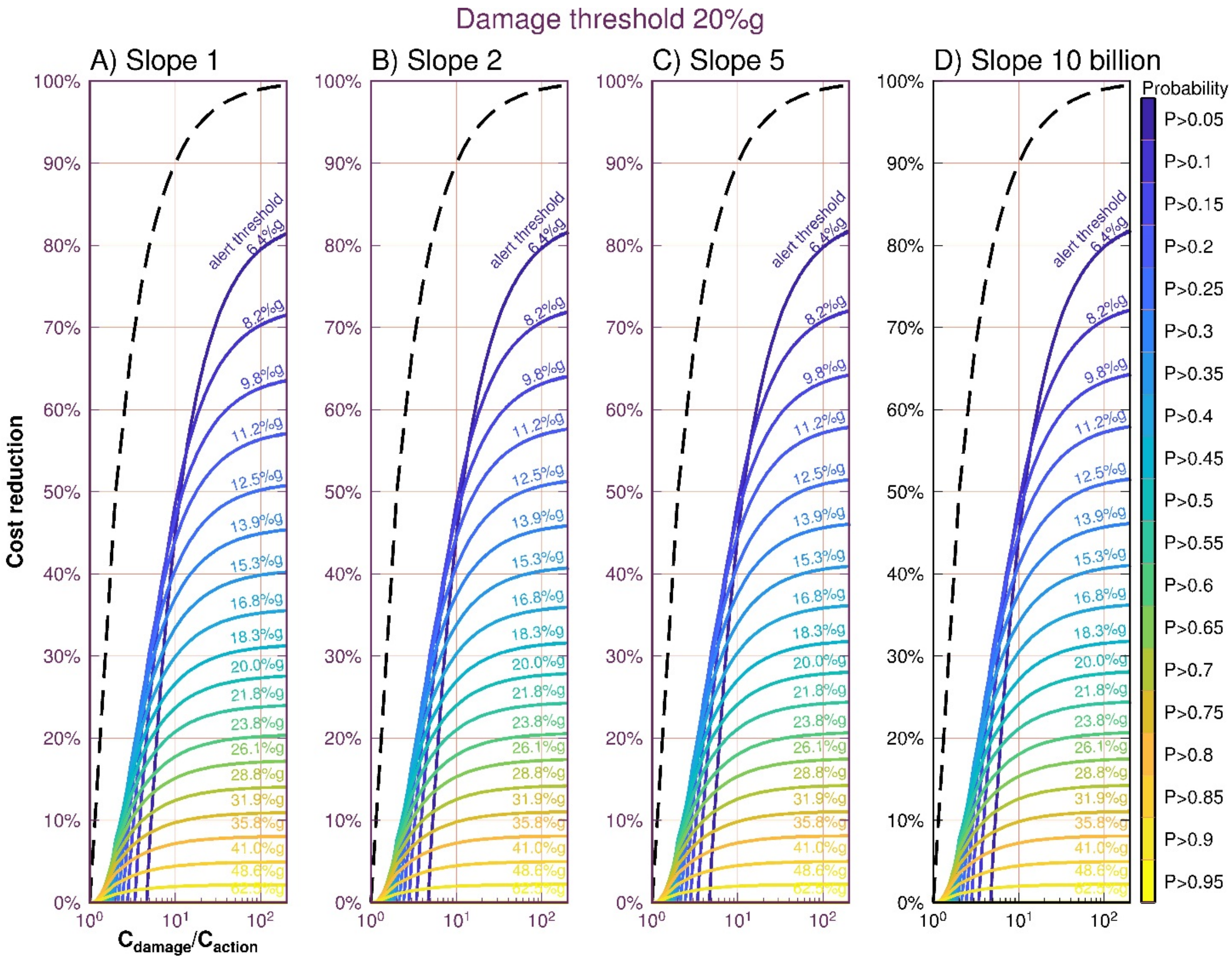


**Fig. S6. Potential cost reduction for different fragility curves centered about 20%*g*.**
Same as Fig. 8(D) for the fragility curves and damage realizations shown in Fig. S4(N-Q). (i.e., the fragility curves are centered about a damage threshold of 20%*g*.) Compare (D) and Fig. 8(D), whose fragility curves are both effectively step functions. Note that while the potential cost reduction increases as the slope steepens, the magnitude of this effect is much smaller than for Fig. S5. This is because the fragility curves are a function of PGA and ground motion variability is a function of log PGA, and thus the magnitude of the effect due to the uncertainty at which ground motion level damage occurs is negligible compared to the effect of ground motion variability at high ground motion levels.

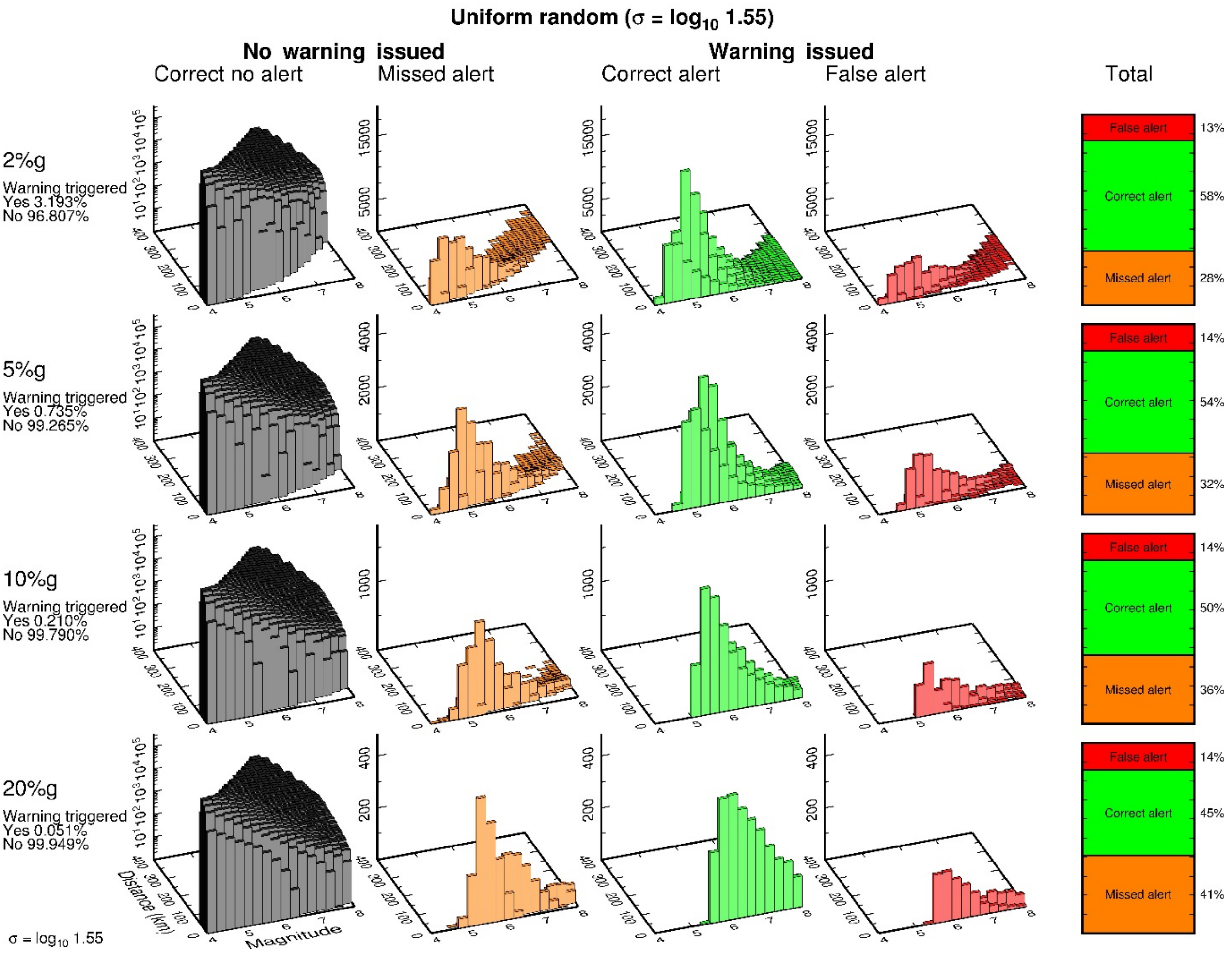


**Fig. S7. Deaggregated earthquake alert outcomes for uniform random catalog assuming smaller uncertainty.**

Same as Fig. 6 but assuming that the ground motion variability of log PGA about the median is log 1.55 (instead of log 2), a typical value of the aleatory within-event variability from regionalized non-ergodic GMPE studies[24,29,30].

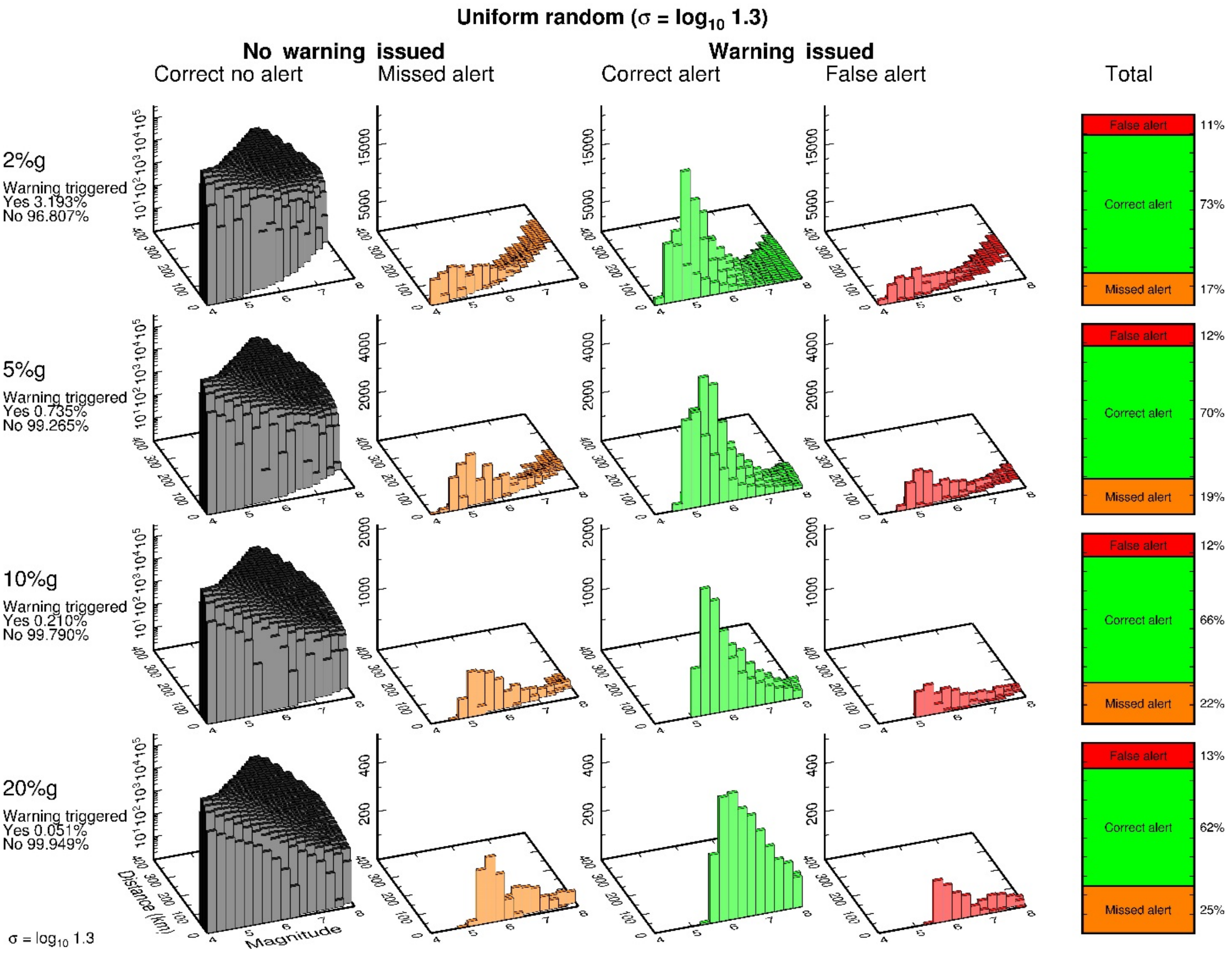


**Fig. S8. Deaggregated earthquake alert outcomes for uniform random catalog assuming even smaller uncertainty.**

Same as Fig. 6 but assuming that the ground motion variability of log PGA about the median is log 1.3 (instead of log 2), an optimistic lower limit on the aleatory within-event variability of GMPEs that represents what we hope to get to with improved completely non-ergodic analysis[31]. This is currently not realized, but represents an ideal GMPE.

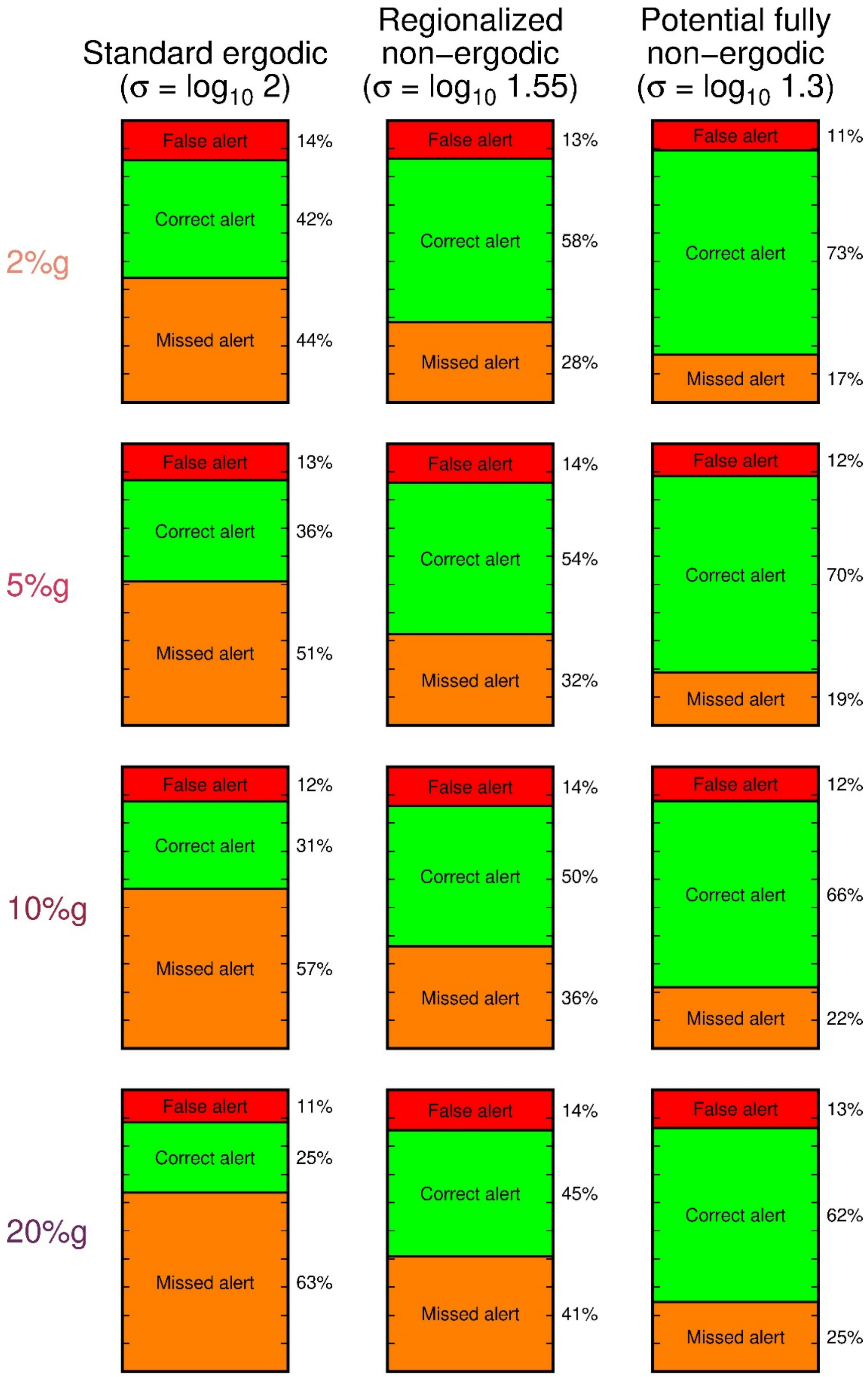
Standard ergodic
(σ = log10 2)
Regionalized non−ergodic
(σ = log10 1.55)
Potential fully non−ergodic
(σ = log10 1.3)
2%g
5%g
10%g
20%g
False alert
Correct alert
Missed alert
14%
42%
44%
13%
58%
28%
11%
73%
17%
13%
36%
51%
14%
54%
32%
12%
70%
19%
12%
31%
57%
14%
50%
36%
12%
66%
22%
11%
25%
63%
14%
45%
41%
13%
62%
25%

**Fig. S9. Comparison of the relative frequency of correct, false, and missed alerts with reduced ground motion variability.**

The results from Fig. 6, Fig. S7, and Fig. S8 are shown in side-by-side comparison. Reducing the total GMPE standard deviation from a factor of 2 (as in currently implemented models) to a factor of 1.3, which we can expect to realize with improved, fully source-, path- and site-specific GMPEs[31] would increase the correct alert rate by as much as ~250% for the largest ground-motion thresholds, while keeping the false alert rate steady, so that missed alerts are reduced accordingly.
